# *Humulus lupulus* L. Strobilus Photosynthetic Capacity and Carbon Assimilation

**DOI:** 10.3390/plants12091816

**Published:** 2023-04-28

**Authors:** William L. Bauerle

**Affiliations:** Department of Horticulture and Landscape Architecture, 1173 Campus Delivery, Fort Collins, CO 80523-1173, USA; bauerle@colostate.edu; Tel.: +1-970-491-4088

**Keywords:** bracts, CO_2_ enrichment, carbon autonomy, flowering crops, lupulin, respiration

## Abstract

The economic value of *Humulus lupulus* L. (hop) is recognized, but the primary metabolism of the hop strobilus has not been quantified in response to elevated CO_2_. The photosynthetic contribution of hop strobili to reproductive effort may be important for growth and crop yield. This component could be useful in hop breeding for enhanced performance in response to environmental signals. The objective of this study was to assess strobilus gas exchange, specifically the response to CO_2_ and light. Hop strobili were measured under controlled environment conditions to assess the organ’s contribution to carbon assimilation and lupulin gland filling during the maturation phase. Leaf defoliation and bract photosynthetic inhibition were deployed to investigate the glandular trichome lupulin carbon source. Strobilus-level physiological response parameters were extrapolated to estimate strobilus-specific carbon budgets under current and future atmospheric CO_2_ conditions. Under ambient atmospheric CO_2_, the strobilus carbon balance was 92% autonomous. Estimated strobilus carbon uptake increased by 21% from 415 to 600 µmol mol^−1^ CO_2_, 14% from 600 to 900 µmol mol^−1^, and another 8%, 4%, and 3% from 900 to 1200, 1500, and 1800 µmol mol^−1^, respectively. We show that photosynthetically active bracts are a major source of carbon assimilation and that leaf defoliation had no effect on lupulin production or strobilus photosynthesis, whereas individual bract photosynthesis was linked to lupulin production. In conclusion, hop strobili can self-generate enough carbon assimilation under elevated CO_2_ conditions to function autonomously, and strobilus bracts are the primary carbon source for lupulin biosynthesis.

## 1. Introduction

The atmospheric CO_2_ concentration is predicted to rise to approximately 500 ppm, reaching 600 ppm and 700 ppm by 2060 and 2075 [1] (pp. 673–816). Elevated atmospheric CO_2_ concentrations can increase a plant’s photosynthesis rate. Most studies of the mechanisms by which plants respond to environmental conditions, such as elevated atmospheric CO_2_, focus on leaves and overlook other green plant organs [2]. It has been shown that extra photosynthetic plant organs, such as the bracts found in abundance in *Humulus lupulus* L. (hop), are a source of assimilated carbon on the ecological and agricultural scales [2,3,4,5,6].

Like leaves, bracts contain chlorophyll and share similarities with leaf morphology and anatomy. However, bract surface area on the plant scale is frequently negligible in comparison to leaves. Hops are an exception, producing upwards of 80 superimposing bracts per strobilus, typically called a hop cone, during the generative growth phase. Each bract on the strobilus is accompanied by two adjacent considerably smaller bracteoles (secondary bracts) [7,8]. The hop bracts and bracteoles’ primary function is to subtend a small flower and glandular trichomes (micro-organs), which are concentrated at the base of each bract. Hop anatomical features are illustrated in Neve [7]. Although a few glandular trichomes exist elsewhere on hop vegetation (e.g., leaves), the bract contains the vast majority. The preponderance of glandular trichomes on bracts indicates that bracts could contribute a significant amount of carbon to their development, analogous to the bracts on cotton bolls [6].

To date, a literature survey reveals a single study on hop strobilus carbon assimilation [9]. Fifty years ago, ambient CO_2_ concentrations were approximately 28% lower, at 325 ppm vs. 416 ppm [1]. Consequently, the extent to which hop bract and bracteole photosynthetic activity responds to current and/or elevated atmospheric CO_2_ is not known. It may be that the strobilus can function autonomously regarding carbon accumulation, where bracts play a significant role in supplying carbohydrate to hop glandular trichomes, commonly known as ‘lupulin glands’. Studies designed to investigate this possibility have not been conducted.

Hop bracts can photosynthesize, and stomata exist on both the adaxial and abaxial surface [9]. The bract surface resembles leaf stoma distribution [10], though approximately 95% fewer stoma exist per surface area in two-sided amphistomatic bract anatomy as compared to on hypostomatic hop leaves [9]. Regardless, the hop bracts that comprise the strobilus contain everything that is of value to beer, predominantly lupulin gland resins, simply termed lupulin. Lupulin consists of a combination of essential oils and alpha and beta acids, which constitute the flavor and fragrance ingredients in beer. The lupulin accumulates during the strobilus maturation stage of the hop fruiting cycle. During this phase, hop strobili amass approximately half of the total aboveground dry matter [7,8]. Strobilus carbon assimilation, crucial to lupulin biosynthesis for beer production, thus warrants further investigation.

Understanding hop strobilus responses to CO_2_ and light would provide insight into their carbon assimilation, autonomy, and response to future and controlled-environment CO_2_ and light conditions. In the present study, we focused on strobilus carbon exchange responses to CO_2_ and light, two growth variables that change spatially and temporally. We hypothesized that under enriched CO_2_ and supplemental light conditions, strobilus function autonomously with respect to carbon assimilation. Simultaneously, we tested a second hypothesis that bracts autonomously generate carbohydrates for lupulin biosynthesis. By isolating individual bract lupulin gland development, bract-generated carbon for lupulin was separated from leaf-derived.

## 2. Results

### 2.1. Strobilus Gas Exchange

#### Net Photosynthesis Versus CO_2_ and Photosynthetically Active Radiation

**Figure 1:**
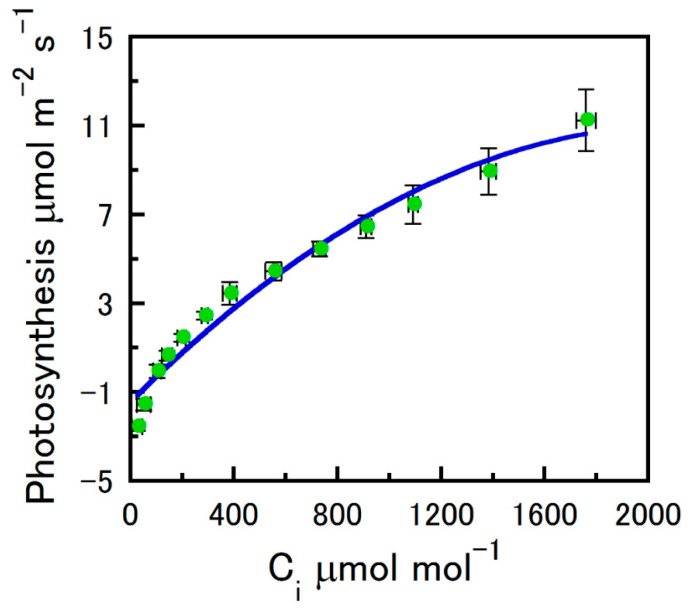
The net photosynthesis of hop strobili as a function of estimated intercellular CO_2_ concentration (C_i_). Cuvette O_2_ was atmospheric ambient (~21%). Strobilus temperature was controlled at 25 °C and photosynthetically active radiation at 400 m^−2^ s^−1^. Samples were pooled across four measurement intervals (means ± SE).

Replicate strobilus net photosynthesis (A_n_) values per intercellular CO_2_ (C_i_) concentration and photosynthetically active radiation (PAR) levels were not different between the four measurement intervals (two-tailed t-tests; *p* > 0.16–0.8 and *p* > 0.23–0.84). Maximum Rubisco carboxylation rate (V_cmax_) and light compensation point (L_c_) values were also stable across the four collection intervals. Figure 1 shows the increase in A_n_, with increases in estimated C_i_ for hop strobili. C_i_ estimates above 1500 μmol mol^−1^ showed increases in A_n_ variation. Nonetheless, strobilus CO_2_ saturation was still not achieved at 1900 ppm atmospheric CO_2_ (Figure 1). Carboxylation limited by the regeneration of inorganic phosphate requires a good estimate of the potential electron transport rate. Due to a lack of CO_2_ saturation in our A_n_/C_i_ response curves, we do not report electron transport (A_nj_) or inorganic phosphate supply (A_np_).

**Figure 2:**
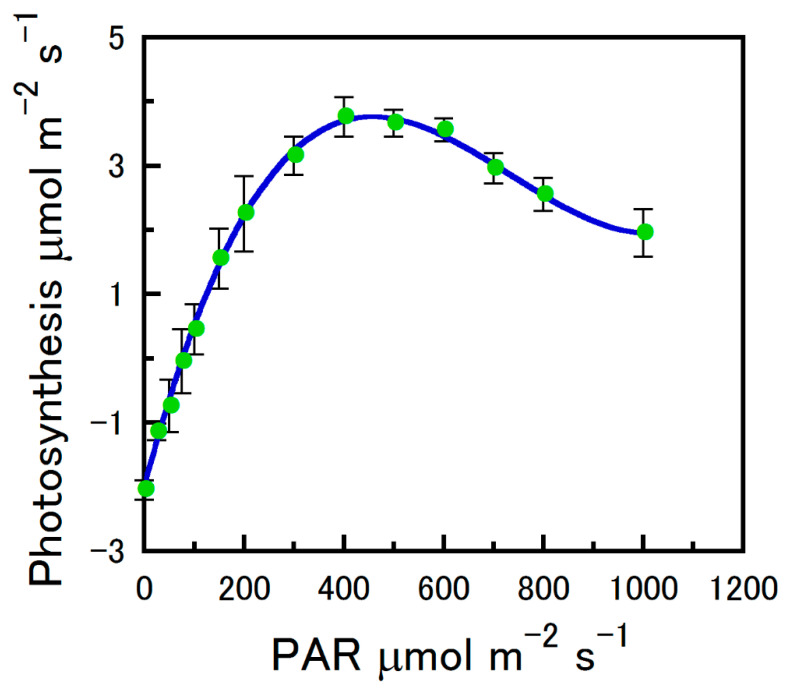
The net photosynthesis of hop strobili as a function of photosynthetically active radiation (PAR). Cuvette O_2_ was atmospheric ambient (~21%). Strobilus temperature was controlled at 25 °C and CO_2_ at approximately atmospheric ambient (415 μmol mol^−1^). Samples were pooled across four measurement intervals (means ± SE).

Compared to leaves, strobilus light saturation (L_s_) occurred at 341 as opposed to 612 µmol m^−2^ s^−1^ (Table 1) [11]. It is probable that high light intensity reduces bract V_cmax_ in hop strobili. In our measurements, PAR above 600 µmol photons m^−2^ s^−1^ reduced A_n_ (Figure 2). Although we avoided this phenomenon by exposing strobili to light saturation at PAR below 600 µmol m^−2^ s^−1^ during the construction of our A_n_/C_i_ curves, supraoptimal light effects could take place in outdoor conditions. For example, 500 µmol m^−2^ s^−1^ PAR represents approximately 25% of the sun intensity on the Earth’s surface. We discovered that approximately 400 µmol m^−2^ s^−1^ PAR is sufficient to achieve strobilus L_s_ and that levels higher that 600 µmol m^−2^ s^−1^ PAR can decrease A_n_ (Figure 2).

We observed a strobilus L_c_ approximately three times higher than that of leaves (76.7 vs. 26 µmol m^−2^ s^−1^ PAR) (Table 1) [11]. Light-saturated photosynthetic rate at ambient atmospheric CO_2_ (415 μmol mol^−1^) (A_max_), on the other hand, was approximately four times lower in strobilus bracts under current ambient CO_2_ concentrations as compared to leaves (4.2 vs. 17.3 µmol m^−2^ s^−1^) (Table 1) [11]. Likewise, strobilus mean quantum yield (ϕ) was half that observed in leaves of the same cultivar (Table 1) [11]. Dark respiration (R_d_) was slightly higher in strobilus as compared to leaf tissue (−2.1 vs. −1.7 µmol m^−2^ s^−1^) (Table 1) [11]. The CO_2_ compensation point (Γ) was higher than values generally observed in C_3_ leaves (Table 1) [11].

### 2.2. Diurnal Carbon Balance Estimates

**Figure 3:**
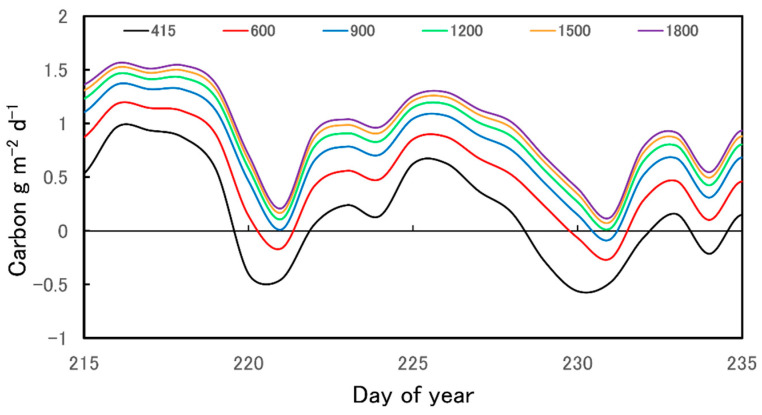
Hop strobili daily carbon gain estimates per m^2^ in Yakima, WA, (representative days of year 215–235, 2022) at ambient (415) and 600, 900, 1200, 1500, and 1800 μmol mol^−1^ CO_2_.

Figure 3 illustrates summed 30 min diurnal strobilus carbon estimates of net daily carbon per strobilus surface area, as described in Section 4.6. Apart from our strobilus gas exchange measurements not encompassing the earliest growth stage, we discovered that strobili exposed to 2022 Yakima, WA, weather variables maintain a close to autonomous carbon balance over the course of 30 days prior to harvest (e.g., Figure 3). On average, strobili generated 92% of their own carbon over the course of 30 days prior to harvest. The daily carbon uptake of strobili exposed to Yakima, WA, 2022 weather conditions at atmospheric CO_2_ of 600 μmol mol^−1^ increased (Figure 3). At 600 μmol mol^−1^, strobili generated 113% of their net carbon balance. Likewise, carbon uptake increased at atmospheric CO_2_ of 900, 1200, 1500, and 1800 μmol mol^−1^ (Figure 3). This resulted in a positive carbon balance of 128%, 136%, 140%, and 143% of net strobilus carbon supply, respectively.

### 2.3. Leaf Defoliation and Bract Carbon Assimilation Interruption

**Figure 4:**
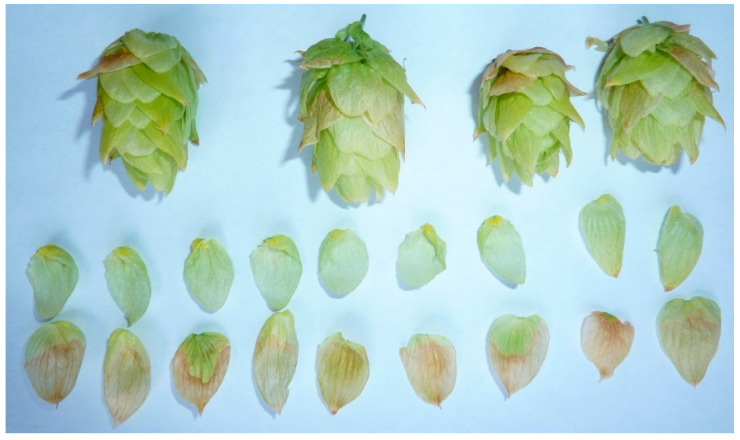
The hop strobili bracts without (**top row**) and with (**bottom row**) contact herbicide treatment. Brown tissue illustrates the contact herbicide treatment and green tissue represents the control. Treated and control bracts on the strobili above the bracts illustrate the random individual treatment of bracts within a strobilus.

**Figure 5:**
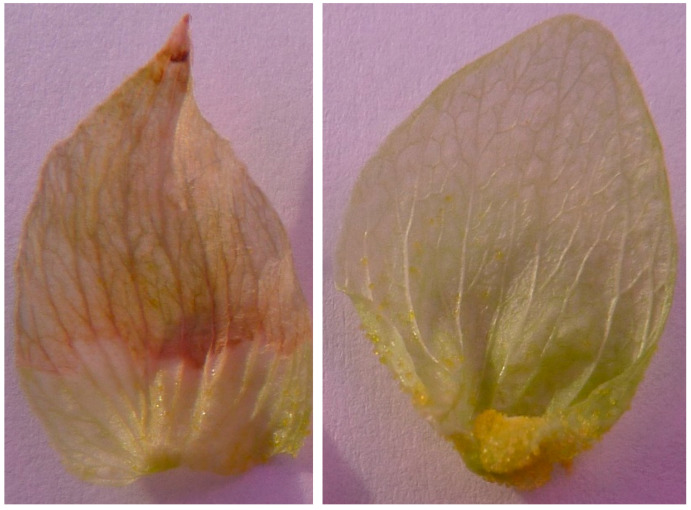
Isolation of hop bracts with (**left**) and without (**right**) contact herbicide treatment. Brown tissue illustrates the contact herbicide treatment and green tissue represents the control. Glandular trichomes are visible at the lower inner surface of each bract. Unlike control bracts, bracts treated with contact herbicide did not fill their glands with yellow lupulin.

To test whether lupulin metabolite production in hops can be explained by a specific photosynthetic source, we removed all the hop leaves after the plants transitioned into reproduction and separately blocked bract photosynthesis from accepting electrons from photosystem I (PSI) via herbicide application. Defoliation did not affect strobilus photosynthetic activity or lupulin production. Blocking bract photosynthesis, to isolate the bract as the sole remaining photosynthetic organ within the strobilus, did prevent lupulin glands from filling. Lupulin glands were amassed approximately equally at the base of each bract in both treatments. Bracts that were not treated yet resided on the same strobilus as some with herbicide treatment filled their basal lupulin glands normally with no visual signs of chlorophyll degradation. Herbicide-treated bracts, however, visually expressed tissue mortality at the site of herbicide contact and failed to fill their lupulin glands (Figure 4). We note that the base of each bract was purposefully not treated with herbicide so as not to injure lupulin glands. Although lupulin glands were present in equal numbers on treated and untreated bracts, glands on bracts treated with herbicide did not fill with lupulin, whereas the glands on untreated bracts of the same strobilus filled (Figure 5). Our results indicate that there is a link between bract photosynthetic electron transport and lupulin biosynthesis, providing indirect support that the bract organ is the primary carbohydrate source of lupulin (Figure 5).

## 3. Discussion

Architecturally and geometrically, the hop strobilus arranges the bract leaf-like organs around a cylindrical axis. The arrangement is one of the most compact that can be achieved when positioning lateral leaf-like organs around a cylindric axis. This configuration not only wholly exposes the strobilus surface area to environmental resources, such as light, but it also allows for two strobilus photosynthesis CO_2_ sources: external atmospheric CO_2_ and internal CO_2_ released from organ respiration. Aschan and Pfanz [12] show that effective internal re-fixation of CO_2_ released from respiratory reactions supplements external atmospheric CO_2_ in extra photosynthetic plant organs [12]. Hop strobili are likely no exception due to the presence of stomata on both the bract adaxial and abaxial side [9]. Respiratory CO_2_ released within the strobilus would result in enrichment above ambient atmospheric conditions, resulting in a shift in the atmospheric CO_2_ to O_2_ ratio, which would reduce the O_2_ inhibition of carboxylation and photorespiration [13,14]. Although bracts possess fewer stomata per unit area compared to leaves [9], their thickness is substantially less than that of leaves. In fact, hop bracts are moist photosynthetic semi-translucent leaf-like structures (Figure 5). CO_2_ is very soluble in water and can quickly diffuse through moist permeable membranes. For example, as compared to other gases with significantly lower molecular weight (e.g., helium or hydrogen) CO_2_’s high solubility in water allows it to diffuse faster through moist membranes.

The net photosynthetic contribution of green reproductive organs to their carbon budget depends on ambient environmental conditions. However, substantial amounts of their carbon requirement can be fixed in situ [15,16]. On a chlorophyll basis, studies on green plant reproductive structures have shown a 1–3 times higher photosynthetic assimilatory capacity as compared to leaves of the same plant species [17,18,19,20,21]. However, the photosynthetic rates and stomatal densities of reproductive organs tend to be lower than those of leaves [2]. In 1970, with ambient atmospheric CO_2_ concentrations of approximately 325 ppm, Peat and Thomas [9] found hop cones ≥ 16 days of age to be capable of partly replacing respiratory losses. Measuring similarly aged strobilus, our A_n_ results match the findings of Peat and Thomas [9]. Measured at current ambient atmospheric concentrations (415 μmol mol^−1^ CO_2_), we found strobili to be capable of counterbalancing 92% of respiratory loss over the course of the month prior to strobilus harvest. When exposed to elevated CO_2_ concentrations, the strobilus carbon budget became positive, thus indicating a strong photosynthetic sugar-producing role in the later stage of the hop strobilus. Similar to prior studies on other reproductive structures, we found that the carbon cost in terms of a strobilus carbon balance may be fully compensated for by increasing photosynthetic rates of the reproductive structure [2,22,23]. Though leaves cannot be ruled out as a carbon source for strobilus development at the onset of the reproductive stage, leaves showed clear signs of senescence during the strobilus generative phase, whereas strobilus photosynthetic capacity did not decline during the hop flowering phase [24]. This indicates that under optimal environmental conditions, strobili can function autonomously with respect to carbon assimilation in the latter phase of the hop growing cycle.

Reproductive structure photosynthesis is common and can provide a source of assimilated carbon on the ecological and agricultural scales [2,3,4,5,6,25]. Under certain environmental conditions, we found strobilus photosynthesis to be capable of counterbalancing respiratory loss, which would decrease the carbon cost of the reproductive structure to the remaining portion of the plant. Moreover, the photosynthesis derived from the strobilus can generate significant amounts of photoassimilates, akin to the large body of evidence for other reproductive organs [2,12]. The hop strobilus is developed in the late stage of the growth cycle. Therefore, its younger photosynthetic machinery can remain photosynthetically active when older leaves have started to senesce (e.g., [26]). Compared to leaves, strobilus PAR L_s_ was >100 μmol m^−2^ s^−1^ lower, whereas the L_c_ was approximately two times higher [11]. Leaf and strobilus respiration rates were similar, but strobilus V_cmax_ decreased by approximately 71% and Γ increased by 43% [11]. Nonetheless, generating a source of carbon from photosynthesis when other source tissue, such as leaves, are senescing allows the strobili to positively contribute to the carbon requirement of reproduction.

Generative-phase defoliated versus leaf-intact plants showed no indication of an influence on trichome gland filling. This suggests that the bracts on strobili can compensate for the loss of sugars from leaves. In contrast, individual bract carbon assimilation interruption versus non-interruption had a clear influence on trichome gland filling. It was proposed that lupulin glands primarily synthesize hop bitter acids [27,28,29]. Due to glandular trichome lupulin accumulation in control bracts and the lack thereof in photosynthetically inhibited bracts, it appears that lupulin biosynthesis may be primarily dependent on bract as opposed to leaf carbon supply. This indicates that glandular trichome lupulin biosynthesis is linked to bract carbon assimilation. It may be that during the generative phase, the hop bine and laterals take on a fundamentally different purpose, mostly functioning as structural support and a pathway for water and nutrient transport [2,30]. In so doing, bine and lateral vegetative structures in the later strobilus development stages would primarily function to spatially disperse strobili throughout the crown to minimize their environmental resource competition, such as light foraging.

The consolidation of carbon-generating and reproductive parts within a strobilus inflorescence may optimize carbon partitioning. In addition, fewer stomata are required due to the higher level of carbon re-fixation from respiratory CO_2_ permeating the bract membrane. In this situation, a reduction in stomatal densities can increase water-use efficiency without a substantial reduction in photosynthetic capacity [31]. Given that hop bracts contain the majority of hop glandular trichomes, it would make sense that bracts provide a portion, if not all, of the carbon to biosynthesize lupulin. In fact, active sinks, such as trichomes, are generally fed by the nearest carbon source [32]. We found that the bract-intensive strobilus possessed a strong capacity to photosynthesize, and provided significant assimilatory products to lupulin under enriched CO_2_ conditions. In addition, strobili bracts showed a lower light saturation as compared to leaves [11]. Future research should investigate strobili chlorophyll fluorescence responses to PAR to possibly explain the observation that supraoptimal PAR lowered A_n_.

In summary, we assessed the link between individual bract photosynthesis and lupulin biosynthesis. The results show unequivocally that the photosynthetic activity of the hop strobilus can maintain a high proportion of the hop plant’s fitness in the later generative phase of the hop. As atmospheric CO_2_ concentrations rise, autonomy of hop strobili could progress further, and one would expect moderate elevations in external CO_2_ concentrations to increase the strobilus carbon supply. However, the carbon gains may be offset by elevated respiration in the face of rising atmospheric temperatures. Nonetheless, our gas exchange results indicate that under current ambient CO_2_ conditions, mature hop strobili can function close to carbon autonomy in the early fall (August) weather of Yakima, WA [33]. Diurnally, the strobilus assimilation gain during daylight nearly counterbalanced the respiratory CO_2_ release in the dark at current ambient CO_2_ concentrations. At CO_2_ concentrations above ambient, the data presented show that ripening hop strobili are capable of a significantly higher proportion of carbohydrate generation.

## 4. Materials and Methods

### 4.1. Plant Material

Research experiments were conducted at the Colorado State University (CSU) Horticulture Center in Fort Collins, Colorado. Over the course of the study, female tissue culture-propagated plantlets were used (Summit Plant Labs Inc., Fort Collins, CO, USA). The public variety ‘Centennial’ was selected due to the representative genetic hop parentage (Brewers Gold + Fuggle + East Kent Golding + Bavarian) [34]. Centennial is ‘ripe to flower’ when ≥ 20 nodes are visible [7,35].

Plantlets were transplanted into 11 L bato buckets containing 100% horticulture-grade perlite. Plant spacing, irrigation, fertilization, and cultural growing condition procedures are outlined in Bauerle [36]. One bine per container was trained to a vertical net trellis at approximately 0.5 m bine length. We note that although there is not a ‘standard’ bine spacing per unit area for hops, the plant density in this study equated to 10,764 bines per ha^−1^, which is a plant density similar to manual field hop production [8]. Initially, all pots were watered to saturation and permitted to drain for 18 h. Thereafter, container moisture capacity was maintained daily. White plastic sheeting was cut and placed on the substrate surface to eliminate evaporation.

### 4.2. Environmental Conditions

Top lights and interlighting bars (suspended horizontally at 150 and 210 cm) (GreenPower LED^®^, Philips Lighting, Amsterdam, The Netherlands) provided supplemental photosynthetically active radiation (PAR) per plant row (100–700 umol m^−2^ s^−1^ during photoperiod). Day length was controlled at 18 and 15 h during the vegetative and generative phases. Controllers were programmed to permit the maximum amount of light penetration (shade cloth was only pulled when intense solar radiation and temperatures demanded additional cooling efforts), and daytime ambient PAR at the canopy surface was generally 800–1100 µmol m^−2^ s^−1^. Greenhouse conditions were programmed to a set point air temperature of 26 °C during the photoperiod and 20 °C during the dark period with a 45 min temperature step change between the two. Daytime temperatures over the experimental period averaged 26.4 °C, but in some instances, temperatures climbed higher despite continuous cooling. Supplemental humidity was provided via an evaporative cooling pad and the daytime saturation vapor pressure deficit (VPD) averaged 1.9 kPa. Air temperature and relative humidity (RH; %) were measured using EHT RH/temperature sensors mounted at the top of the canopy (Meter Devices, Pullman, WA, USA) and PAR using two quantum line sensors placed parallel to the north/south row orientation adjacent to the plant stems (model LI191R, Li-Cor Inc., Lincoln, NE, USA). A third quantum line sensor was placed 0.5 m above the canopy. The line sensors sampled PAR every minute and then recorded a 15 min average (CR10x; Campbell Scientific, Logan, UT, USA).

### 4.3. Strobilus Gas Exchange and Light Absorption Measurements

To examine the photosynthetic capacity characteristics, six replicate plants, located in the center of the plot, were sampled for strobilus gas exchange traits. Sixteen days after anthesis, ten-day interval repeated measurements began on fully expanded strobili using a portable gas exchange system (CIRAS-2, PP Systems, Haverhill, MA, USA). A PP Systems environmentally controlled conifer branchlet cuvette (Model PLC5 (C)) was used to measure whole strobili. PAR was controlled with a full-spectrum light-emitting diode and temperature by means of Peltier elements fitted with heat sinks and fans (Model PLC5 (C), PP Systems, Haverhill, MA, USA). The measurements were performed at a controlled VPD of 1.5 kPa and temperature of 25 °C. A preliminary experiment indicated A_n_ stability after 10 min per CO_2_ change and five minutes per PAR change.

Response curves of net photosynthesis (A_n_) versus [CO_2_] (A_n_/C_i_) and PAR (A_n_/PAR) were repeated four times at ten-day intervals. For the A_n_/C_i_ curves, strobili were initially acclimated in the chamber until A_n_ was stable at a controlled CO_2_ concentration (415 μmol mol^−1^) and light level (400 μmol m^−2^ s^−1^). The CO_2_ response curve was constructed at CO_2_ levels of 415, 300, 200, 150, 100, 50, 25, 415, 600, 800, 1000, 1200, 1500, and 1900 μmol CO_2_ mol^−1^ with 10 min of stabilization per CO_2_ change. After the completion of the A_n_/C_i_ curve, CO_2_ concentration was controlled at 415 μmol mol^−1^ until stabilization, and PAR was sequentially lowered to 1000, 800, 700, 600, 500, 400, 300, 200, 150, 100, 75, 50, 25, and 0 μmol m^−2^ s^−1^ with five minutes of stabilization per PAR change.

Immediately after gas exchange, five replicate light absorptance samples were recorded per strobilus on random bracts within the strobilus with an SPAD meter and averaged (model 502B, Minolta Inc., Ramsey, NJ, USA). The SPAD values were used to correct apparent quantum yield (ϕ_a_) to ϕ, as described in Bauerle et al. [37]. For photosynthesis calculations, surface bract area (BA) per hop strobilus was measured and calculated as the total surface area of a cone:

BA = πr (r + l),(1)

where radius (r) and slant height (l) account for the area of the cylinder base and the cone. (R^2^ = 0.968, *n* = 5; unpublished data).

### 4.4. A_n_/C_i_ Curve Fitting

The model of Farquhar, von Caemmerer, and Berry [38] was used to estimate the maximum Rubisco carboxylation rate (V_cmax_) under light saturation due to the capability of estimating in vivo Rubisco activity. The A_n_/C_i_ data were analyzed as per the fitting method “fitaci” function of the “plantecophys” package [39] according to Wullschelger [40], where the minimum of any of the three factors Rubisco activity (A_nc_), electron transport (A_nj_), and inorganic phosphate supply (A_np_) can limit CO_2_ assimilation, as summarized in von Caemmerer et al. [41]

A_n_ = min (A_nc_, A_nj_, A_np_), (2)

The partial pressure of CO_2_ in the chloroplast at which photorespiratory CO_2_ evolution equals the rate of carboxylation (Γ*; 38.6 umol m^−2^ s^−1^) at 25 °C and 21% O_2_ was as in von Caemmerer et al. [42], assuming an average atmospheric pressure of 847 mbar in Fort Collins, Colorado. The Γ was calculated from the fitted curve.

### 4.5. A_n_/PAR Curve Fitting

Photosynthesis versus PAR data were fitted to the nonrectangular hyperbola model of Prioul and Chartier [43], as described in Parsons et al. [44]

(3) $$A_{n}=\phi_{a}\;\mathrm{PAR}+A_{\max}-\surd (\phi_{a}\;\mathrm{PAR}+A_{\max}{)}^{2}-4\;\phi_{a}\;\mathrm{PAR}\;ø\;A_{\max}/2ø-R_{d}$$

where A_max_ is the light-saturated net photosynthetic rate, ϕ_a_ is the apparent quantum yield of assimilation, ø is the convexity of the curve, and R_d_ is the respiration rate. The parameter ϕ_a_ was calculated through linear regression analysis on the initial slope from 20–125 μmol m^−2^ s^−1^ to exclude the Kok effect region (≤20 μmol m^−2^ s^−1^) [45] and prevent ϕ_a_ underestimation from data in the non-linear region (≥125 μmol m^−2^ s^−1^) [46]. The light compensation point (Q_c_) was estimated from the intersection of the regression line with the x-axis (A_n_ = 0), and R_d_ was measured at the end of the A_n_/PAR curve. The quantum yield for CO_2_ (ϕCO_2_) was derived from ϕ_a_ by correcting ϕ_a_ for the percentage leaf absorptance of PAR, as described in Bauerle et al. [29]. Equation (3) was fitted separately for the independent A_n_/PAR curves (Photosyn Assistant, Ver. 1.1.2, Dundee Scientific, Dundee, UK).

### 4.6. Diurnal Strobilus Carbon Balance Estimates

Photosynthetic light response and A_n_/C_i_ parameters were used to parameterize deterministic strobilus-level models for energy balance [47], stomatal conductance (e.g., [48,49]), and photosynthesis [38] (Table 1). A simulation of carbon gain was performed at light saturation to estimate the impacts of atmospheric elevated CO_2_. To place the strobilus CO_2_ responses in the context of strobilus diurnal carbon gain, the photoperiod was set at 15 h, the approximate day length for strobilus physiological functions in Yakima, WA, (46.6021° N, 120.5059° W) at the beginning of August (day of year 213). Half-hourly averages of incident PAR, temperature, RH, and wind speed in Yakima, WA, were downloaded from the Washington State University AgWeatherNet site. Atmospheric CO_2_ concentrations of 415, 600, 900, 1200, 1500, and 1800 μmol mol^−1^ were inputted to estimate strobilus carbon gain responses to CO_2_ enrichment.

Predicted strobilus carbon gain (C_g_) is a direct estimate of carbohydrate biosynthesis:

C_g_ = (A_n_;light − R;dark) × 12, (4)

where A_n_;light is the total A_n_ during the light period, R;dark is the total respiration during the dark period, and 12 is the molecular mass of C. We summed 30 min diurnal carbon estimates to arrive at net daily C_g_ per strobilus surface area.

### 4.7. Bine Defoliation and Bract Photosynthesis Interruption

To investigate the carbon source for lupulin biosynthesis on the plant scale, six replicates were randomly assigned to either the “defoliation” or “control” group. The control was left to grow normally, and the defoliation group had all leaves removed using scissors at the strobili stigma senescence stage. The strobili stigma senescence stage, described in the comprehensive strobilus developmental stage index of Kavalier et al. [50], is informative because it occurs in advance of biosecratory lupulin production (gland filling) [50]. On the strobilus scale, a photosynthetic inhibitor was used to assess the contribution of bract photosynthesis to lupulin biosynthesis. Paraquat (1,1′-dimethyl-4,4′-bipyridylium), an herbicide absorbed very quickly by leaves, was applied to random bracts at a concentration of 250 g/L via CCT2425 swabs (Chemtronics^®^ Kennesaw, GA, USA) to inhibit electron transport and CO_2_ assimilation. Per strobilus, several random bracts were swabbed with Paraquat from the bract tip proceeding inward toward the strig, covering approximately two thirds of the top surface of an individual bract. Six replicate plants each per defoliation or control group were treated. Strobili were harvested upon maturity and lupulin gland secretions were visually assessed at 40× magnification on Paraquat-treated and untreated bracts per strobilus.

### 4.8. Statistical Analysis

The total sample size for A_n_/PAR and A_n_/C_i_ curves was six replicates (*n* = 6), with one strobilus per six replicate plants. Each strobilus was repeatedly measured at ten-day intervals over the course of four repeated measures (40 days). Each plant was an experimental unit treated as a replicate (*n* = 6). Two-tailed *t*-tests were used to analyze the significance between each combination of repeated time measurements. Plant response data were analyzed using SPSS (IBM Analytics, Armonk, NY, USA, www.ibm.com/analytics/ (accessed on 7 November 2022)). Differences between means were considered significant when the *p* value of the *t*-value was < 0.05.

## Figures and Tables

**Table 1:** Photosynthetic response parameters for hop strobili of cv. ‘Centennial’ measured at 25 °C. Means ± standard error (SE). Photosynthetically active radiation response parameters are light-saturated photosynthetic rate at 415 μmol mol^−1^ CO_2_ (A_max_, µmol m^−2^ s^−1^), dark respiration rate (R_d_, µmol m^−2^ s^−1^), quantum yield (ϕ, mol C mol^−1^), and light saturation point (L_s_, µmol m^−2^ s^−1^) at 415 μmol mol^−1^ and 25 °C. CO_2_ response parameters are maximum Rubisco carboxylation rate (V_cmax_) (μmol m^−2^ s^−1^) and CO_2_ compensation point (Γ, μmol mol^−1^) at 25 °C. Samples were pooled across four measurement intervals. Number of strobili *n* = 24.

Parameter	Mean ± SE
R_d_	−2.1 ± 0.3
A_max_	4.2 ± 0.3
ϕ	0.033 ± 0.002
L_s_	341.0 ± 16.5
L_c_	76.7 ± 12.6
V_cmax_	14.1 ± 1.1
Γ	110.3 ± 13.3

## Data Availability

The data presented in this study are available on request from the corresponding author.
